# Correlation Between Vitreous Level of Angiogenic Growth Factors and Oxygen Saturation in Retinal Vessels in Diabetic Retinopathy

**DOI:** 10.1167/iovs.64.15.4

**Published:** 2023-12-04

**Authors:** Hana Morin, Jan Havlík, Oldřich Chrapek, Michal Hrevuš, Pavel Němec, Leoš Rejmont, Jan Tesař, Marta Kalousova, Tomaš Zima, Martin Šín

**Affiliations:** 1 Department of Ophthalmology, Military University Hospital Prague, 1^st^Faculty of Medicine, Charles University, Prague, Czech Republic; 2 Department of Ophthalmology, Faculty of Medicine, Masaryk University Brno, Czech Republic; 3 Department of Ophthalmology, University Hospital, Brno, Czech Republic; 4 Department of Ophthalmology, University Hospital Olomouc, Faculty of Medicine and Dentistry, Palacký University Olomouc, Olomouc, Czech Republic; 5 Institute of Medical Biochemistry and Laboratory Diagnostics, First Faculty of Medicine, Charles University, and General University Hospital in Prague, Prague, Czech Republic

**Keywords:** retinal oximetry, hypoxia, diabetic retinopathy, vascular endothelial growth factor

## Abstract

**Purpose:**

The aim of this study was to investigate whether there is any association between the levels of the angiogenic growth factors and the vascular oxygen saturation in eyes with diabetic retinopathy.

**Methods:**

The study was designed as a prospective trial. The cohort consisted of 29 diabetic patients with scheduled vitreous procedures (intravitreal injection or pars plana vitrectomy). The control group included 30 patients scheduled for macular surgery (macular hole or epiretinal membrane). Nine patients (four from the diabetic maculopathy [DM] group and five from the control group) were excluded from the study because of unsuccessful vitreous samples. Retinal oximetry was performed several hours before the vitreous procedure was performed, and vitreous samples were obtained during the procedure. The concentrations of VEGF, Serpin F1/pigment epithelium–derived factor (PEDF), and placental growth factor (PlGF) were measured by ELISA.

**Results:**

A negative correlation between level of VEGF and arteriovenous (AV) saturation difference was determined in the DM group (Pearson correlation coefficient *r* = −0.607; two-tailed test, *P* = 0.002). Also a negative correlation between level of PlGF and AV saturation difference was determined in the DM group (Pearson correlation coefficient *r* = −0.521; two-tailed test, *P* = 0.011) A positive correlation between PlGF level and the vein saturation was not statistically significant (Pearson correlation coefficient *r* = 0.325; two-tailed test, *P* = 0.130). We did not find any correlation between vitreous level of PEDF and vascular saturation within the DM group.

**Conclusions:**

Our findings in diabetic patients suggests a correlation between the intravitreal level of proangiogenic factors and the AV difference measured by retinal oximetry.

Over the past decade, several studies have shown changes in oxygen saturation in the retinal vessels of diabetic retinopathy (DR).^1^^–^^6^ An increase in venous oxygen saturation in retinal vessels and corresponding decrease of the arteriovenous (AV) difference was shown to be related to the severity of the retinopathy.^1^^,^^4^^–^^6^ In studies where hypoxia was expressed by the Ischemic Index in DR^7^ and, similarly, in retinal vein occlusion cases,^8^ a relationship to changes of O_2_ saturation in retinal vessels was found. Hypoxia in turn causes an increased synthesis of angiogenic growth factors, mainly the VEGF. VEGF is the most potent vasoactive factor, and its normal expression is necessary for maintaining the structural and functional homeostasis of retinal cells. However, the overexpression of VEGF because of pathological factors such as hypoxia and hyperglycemia could lead to abnormal retinal angiogenesis.^9^

Our literature search revealed a paucity of studies on the association of vitreous VEGF levels and the changes of saturation in retinal vessels of DR patients. In our current study, we have investigated whether there is any association between the levels of the most potent angiogenic growth factors and the vascular oxygen saturation in eyes with DR.

## Materials and Methods

We performed a two-center prospective study to investigate the relationship between the vitreous levels of angiogenic growth factors and oxygen saturation within retinal vessels in diabetic patients. The study protocol was approved by the Ethics Committee of the University Military Hospital in Prague (protocol no. 108/16-82/2021) and the University Hospital in Brno, Czech Republic (protocol no. 02-160222/EK). The study was implemented in accordance with Good Clinical Practice and the Declaration of Helsinki. All patients provided written informed consent before enrollment into the study.

The sample consisted of 29 eyes in 29 diabetic patients with scheduled vitreous procedures (intravitreal injection or pars plana vitrectomy [PPV]). Exclusion criteria were patients with a history of retinal vascular occlusion, glaucoma, and AMD (i.e., ischemic ocular conditions that could potentially skew the results), patients with a media opacity such as dense cataracts or severe vitreous hemorrhage (i.e., conditions that could obscure retinal oximetry images or retinal photographs), and patients who have undergone panretinal photocoagulation. Patients with systemic conditions such as severe respiratory disease (e.g., chronic obstructive pulmonary disease), severe anemia, or sickle cell disease that could affect the retinal oximetry results were also excluded. The control group represented 30 eyes of 30 patients scheduled for macular surgery (PPV for macular hole [MH] or epiretinal membrane [ERM]). In all cases, retinal oximetry was performed several hours before the vitreous procedure (range one to 12 hours) and vitreous samples were obtained during the procedure. Nine patients (four from the diabetic maculopathy [DM] group and five from the control group) were excluded from the study because of unsuccessful vitreous samples (dry samples), and two patients in the DM group were excluded based on insufficient oximetry image quality. A total of 50 patients (25 in the control group and 25 in the DM group) entered the final evaluation in this study. Enrollment of patients was carried out consecutively between November 2021 and June 2022.

In the DM group, the average age was 62 ± 14 years, and the male-to-female ratio was 14/11. In this group, three patients had DM with no sign of DR and were scheduled for PPV with ERM. Eight patients had moderate nonproliferative DR (NPDR) with diabetic macular edema (DME), seven patients had severe NPDR with DME and were scheduled for anti-VEGF treatment, and seven had proliferative DR (PDR) and were scheduled for PPV. In the control group, the average age was 72 ± 8 years with nine male and 16 female patients. All demographic data are presented in Table 1.

**Table 1.** Demographic Data

Group	Diabetic	Control	*P* Value
Number of enrolled	29	30	
Excluded from analysis	4	5	
Suitable for analysis	25	25	
Age	62 ± 14	72 ± 8	**0.0056**
Male/female ratio	11/14	9/16	NA
Arterial saturation (%)	100% ± 6%	94% ± 6%	**0.0060**
Vein saturation (%)	65% ± 9%	58% ± 12%	**0.0246**
AV difference (%)	35% ± 7%	37% ± 7%	0.2828
VEGF (pg/mL)	388 ± 683	BDL	**0.0159**
PlGF (pg/mL)	46 ± 71	BDL	0.0552
PEDF (ug/mL)	12 ± 9	8 ± 4	0.0517

*P* values in bold are statistically significant.

### Automatic Retinal Oximetry

The Oxymap T1 retinal oximeter (Oxymap Inc, Reykjavik, Iceland) was used in the study. The Oxymap T1 is attached to the view port of a fundus camera (Topcon DX-50; Topcon Inc, Tokyo, Japan) and measures hemoglobin oxygen saturation in retinal vessels using the ratio of light absorbance at 600 nm and 570 nm (OD600/OD570). Details of the device are described in other publications.^10^ The oximetry software detects retinal vessels and defines pixels as either belonging or not belonging to a vessel. The software estimates the vessel diameter at a particular point by counting the number of pixels, defined as vessel pixels, on an orthogonal cross-section of the vessel. These values are then averaged along the measured vessel for more than 100 cross-sections to give the vessel diameter. A previous study has validated this approach.^11^ Vessels that measured below 8.0 pixels (approximately 75 nm) in diameter were excluded from the calculation of the mean value for the respective eye. The retinal oximeter is calibrated to give hemoglobin oxygen saturation values that are both repeatable and sensitive to changes in hemoglobin oxygen saturation.^12^ Because T1 is calibrated for nondiabetic young individuals, results are relative to that calibration, occasionally resulting in arterial saturation greater than 100%.^7^ A standardized technique for all measurements was used. In brief, we used a darkroom to avoid the influence of light.^13^ Fundus photographs in a 50° field were focused on the temporal edge of the optic disc and we set the light flash at 50 Ws. The snapshot was performed after an approximate delay of five seconds, with the camera focusing light being switched off.

### Automatic Retinal Oximetry Analysis

**Figure 1.**
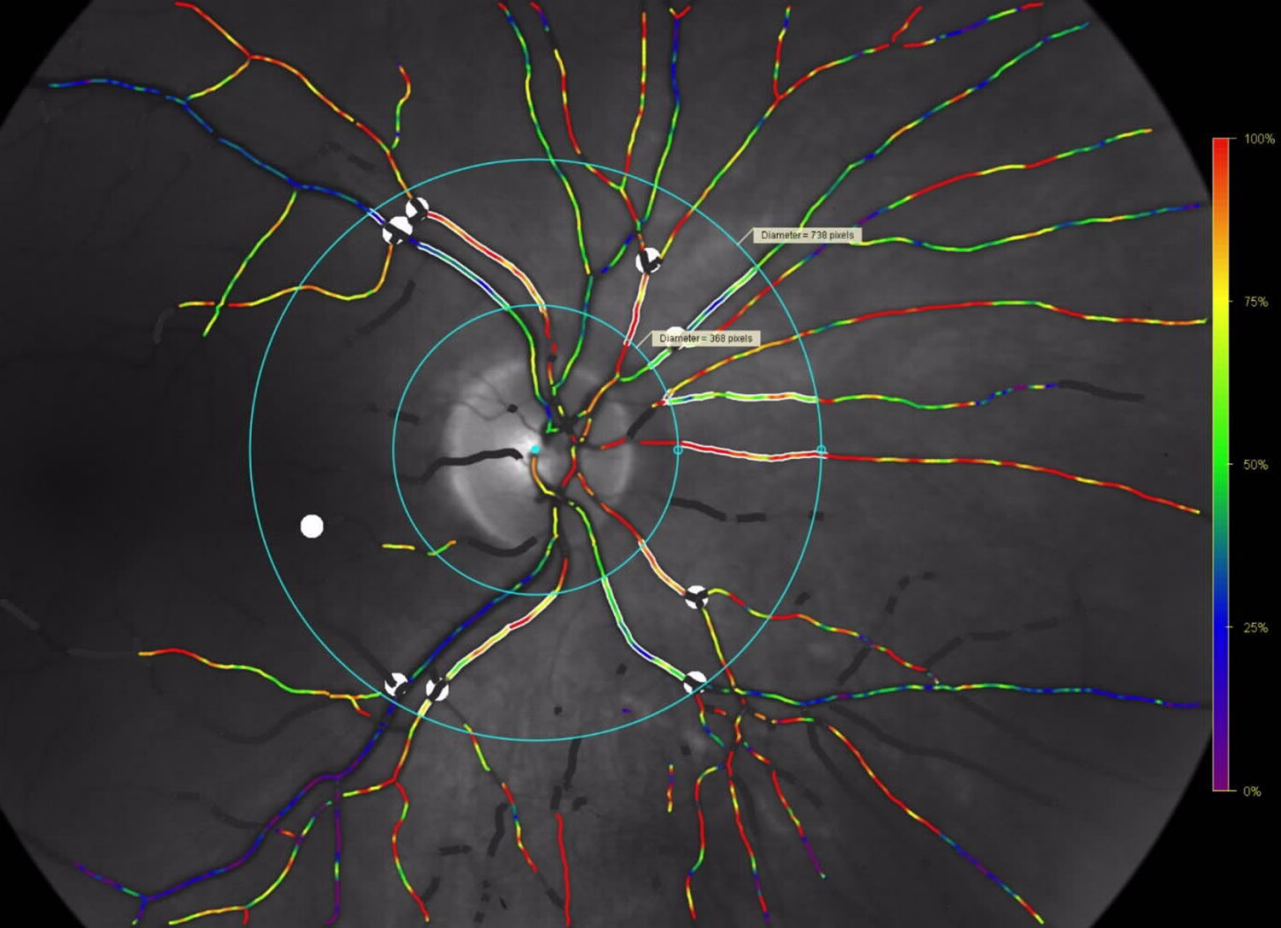
An oximetry image. The two *blue circles* demarcate the measurement area. The *inner circle* has a diameter of 1.5 disc diameter. The *outer circle* has a diameter of 3.0 disc diameters. The main retinal vessel segments in this area are chosen according to a set of detailed rules (Oxymap protocol from November 21, 2013, simple means used).

Quality of the fundus photographs was evaluated, and only image quality with values over 5.0 out of 10.0 were considered eligible for the study. Segments of first- and second-order arteries and veins longer than 100 pixels (approximately 0.9 mm) and located in the measuring area were selected for oxygen saturation analysis. The mean saturation over the vessel segment was calculated. The measuring area was defined between a circle located on the edge of the optic disc and a second circle three times the diameter of the optic disc circle (Fig. 1)*.* Vessels in all quadrants were used. The difference between arterial and venous oxygen saturation was calculated and represented as an arteriovenous oxygen difference (AV difference). For all the analyses, we used Oxymap software (version 2.5.2; Oxymap Inc, Reykjavik, Iceland). The analysis was performed by a single masked examiner.

### Vitreous Samples

Vitreous samples were collected either by vitreous tap or biopsy before PPV. Sampling was done by insertion of a 27 gauge needle attached to a 1 mL tuberculin syringe through the 25 gauge port in the pars plana region into the vitreous cavity and aspiration of 0.1 to 0.2 mL of vitreous fluid was done. A total of 50 samples were obtained. Fifteen of these samples were patients scheduled for anti-VEGF treatment, and 10 cases were patients with ERM scheduled for a PPV. For controls, we used 25 patients scheduled for PPV with idiopathic macular hole (IMH) or ERM served. The vitreous samples were stored at −80°C immediately after the sampling procedure.

### Laboratory Analysis of Vitreous Samples

The concentrations of VEGF, serin proteinase inhibitor–clade F1/pigment epithelium-derived factor (Serpin F1/PEDF), and placental growth factor (PlGF) were measured by ELISA using commercial kits (R&D Systems, Minneapolis, MN, USA) according to the manufacturer's protocol. Sensitivity of assays was 9 pg/mL for VEGF, 7 pg/mL for PlGF, and limit of quantification of the Seprin F1/PEDF ELISA test developed from DuoSet kit was 0.78 µg/mL.

### Statistical Analysis

All statistical analyses were performed using R version 4.2.2 (The R Foundation for Statistical Computing). First, we tried to find a statistical correlation between measurements related to retinal vessel saturation and levels of growth factors. Based on the significance of the correlation coefficient, we selected the pair of AV difference and VEGF and the pair of AV difference and PlGF for further analysis. We then performed a normality test (Daniel-Anderson) of the AV difference and VEGF and PlGF. Because the AV difference passed the test, the hypothesis of normality for VEGF and PlGF was rejected. After applying the log(VEGF) transformation, the transformed data were tested for normality. We then used AV difference and log(VEGF) for subsequent analysis. The data showed a significant linear correlation for the DM group, which was verified by the correlation coefficient significance test. The significance of the difference in the averages of the measured values for the “DM” and “Control” groups was verified by a two-sample *t*-test. The data are presented as a mean ± standard deviation (unless indicated otherwise), and the difference at *P* < 0.05 was considered significant.

## Results

**Figure 2.**
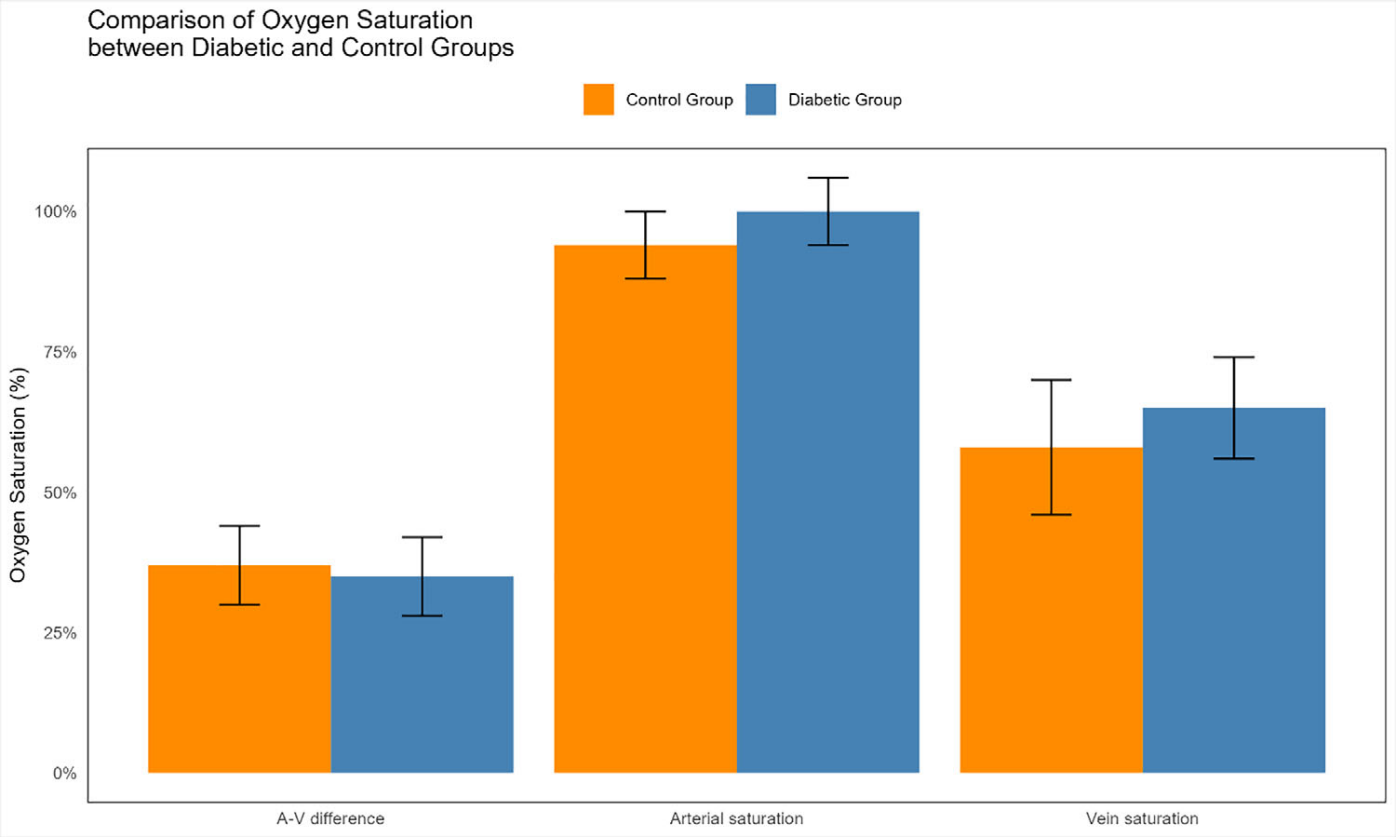
Bar charts of retinal vessel oxygen saturation values in the different study groups.

In eyes in the DM group, the mean arterial saturation was 100% ± 6%, the mean vein saturation was 65% ± 9%, and the mean AV difference was 35% ± 7%. In the control group, the mean arterial saturation was 94% ± 6%, the mean vein saturation was 58% ± 12%, and the mean AV difference was 37% ± 7% (Fig. 2).

**Figure 3.**
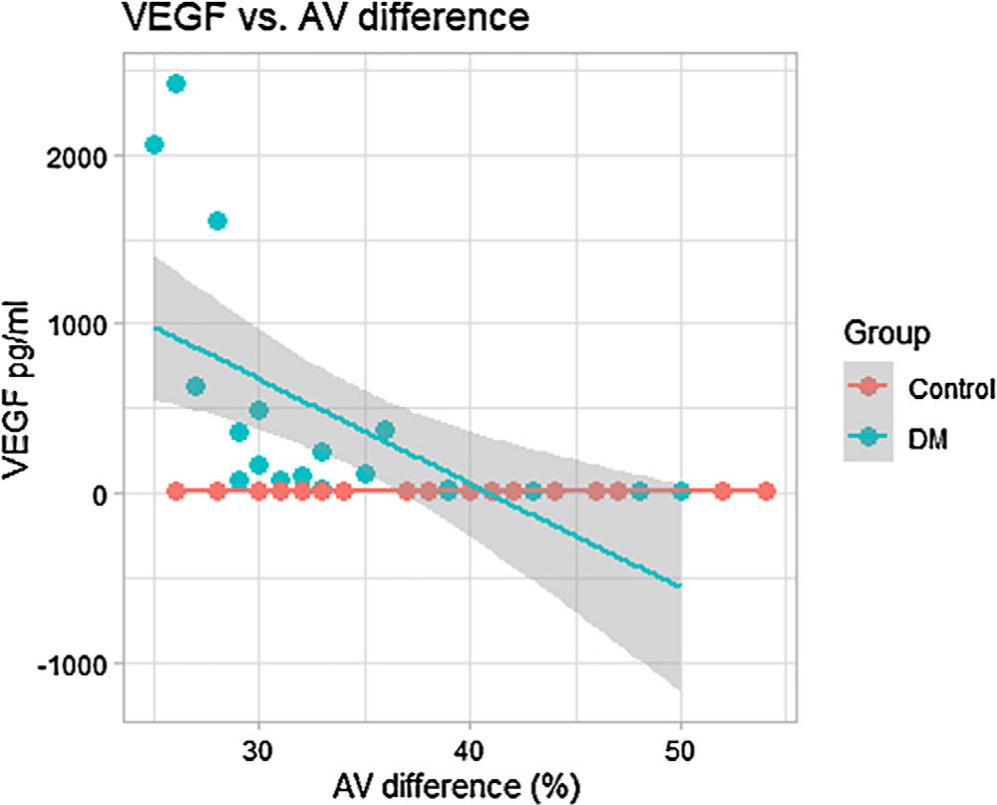
Correlation between AV difference (%) and vitreous level of VEGF (pg/mL). Pearson’s correlation coefficient *r* = −0.607; two-tailed test, *P* = 0.002.

A negative correlation between vitreous level of VEGF and AV saturation difference was determined in the DM group (Pearson correlation coefficient *r* = −0.607; two-tailed test, *P* = 0.002) (Fig. 3). A positive correlation between vitreous VEGF level and the vein saturation was on the edge of statistical significance in the DM group (Pearson correlation coefficient *r* = 0.386; two-tailed test, *P* = 0.069).

**Figure 4.**
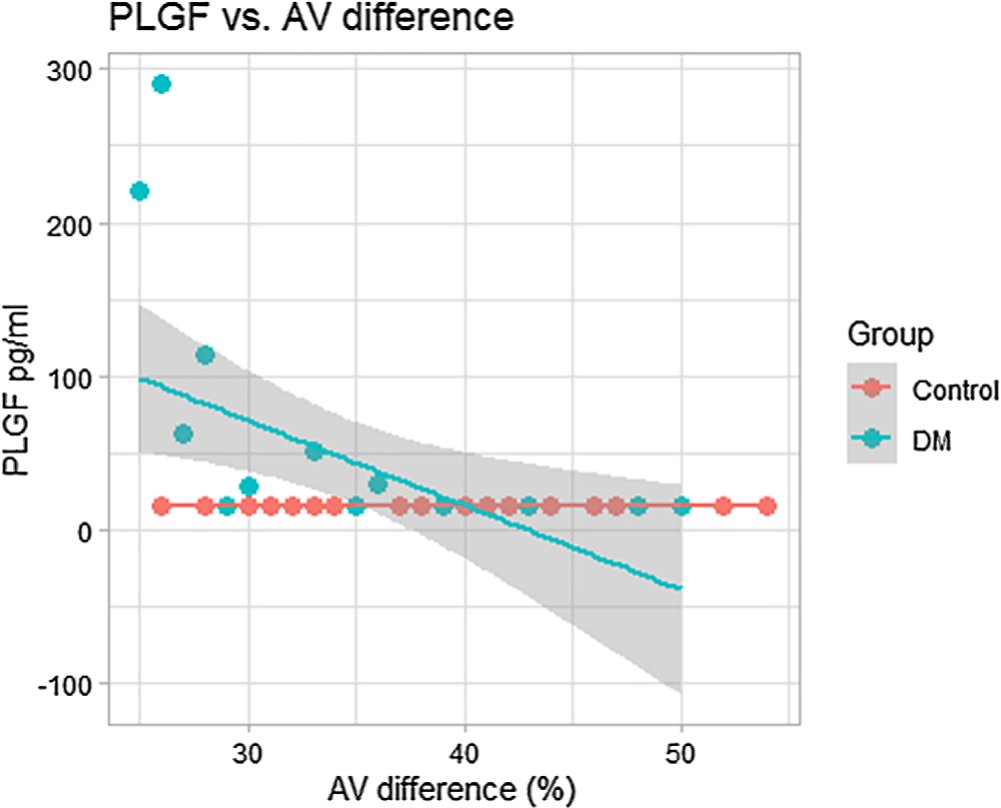
Correlation between AV difference (%) and vitreous level of PlGF (pg/mL). Pearson’s correlation coefficient *r* = −0.521; two-tailed test, *P* = 0.011.

A negative correlation between vitreous level of PlGF and AV saturation difference was determined in the DM group (Pearson correlation coefficient *r* = −0.521; two-tailed test, *P* = 0.011) (Fig. 4). A positive correlation between vitreous PlGF level and the vein saturation was not statistically significant (Pearson correlation coefficient *r* = 0.325; two-tailed test, *P* = 0.130).

**Figure 5.**
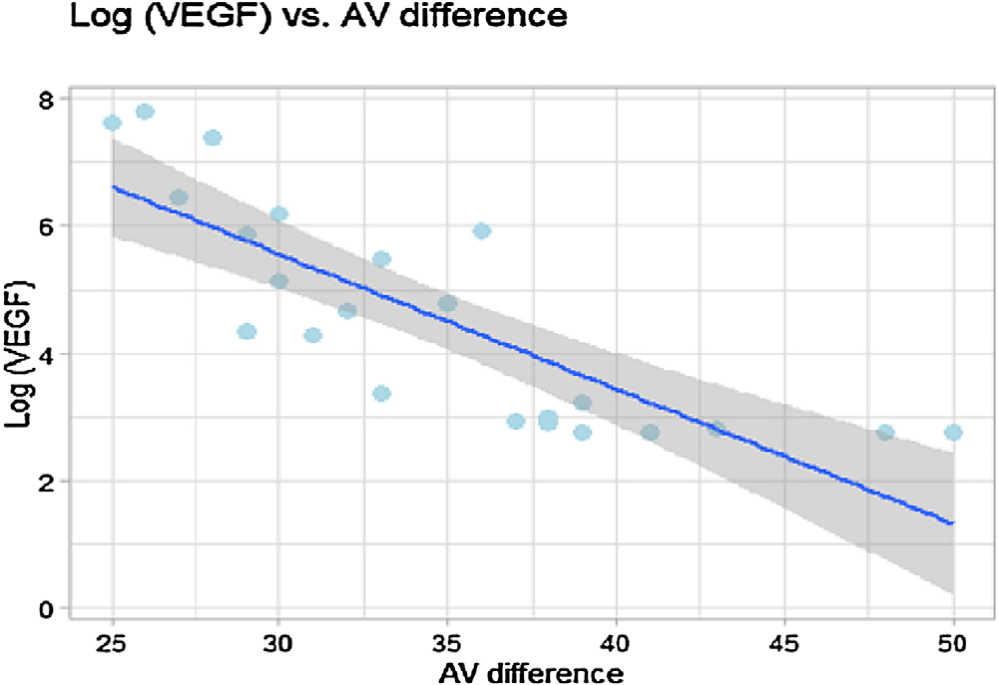
Correlation between AV difference (%) and log VEGF values. Pearson’s correlation coefficient *r* = −0.821; two-tailed test, *P* < 0.00001.

A negative correlation between AV saturation difference and logVEGF data was determined for the DM group (Pearson correlation coefficient *r* = −0.8205; two-tailed test, *P* < 0.00001) (Fig. 5).

We did not find any correlation between vitreous level of PEDF and retinal vascular saturation within the DM group. Table 2 shows all correlations among vitreous levels of angiogenic factors and retinal vascular saturation values in the DM group.

**Table 2.** Pearson's Correlation for the Diabetic Group

Saturation	Growth Factor	Correlation	*P* Value
AV difference	VEGF	−0.607	**0.0021**
AV difference	PLGF	−0.521	**0.0107**
Arterial saturation	PEDF	−0.389	0.0664
Vein saturation	VEGF	0.386	0.0687
Vein saturation	PLGF	0.325	0.1299
Vein saturation	PEDF	−0.218	0.3173
Arterial saturation	PLGF	−0.099	0.6519
Arterial saturation	VEGF	−0.098	0.6559
AV difference	PEDF	−0.073	0.7400

## Discussion

In our current study, the findings indicate a statistically significant increase in arterial and venous saturation in patients with diabetes. Corresponding decrease of AV difference (from 37% to 35%) did not reach statistical significance. There is still some controversy between the finding of decreasing AV difference with progressing severity of diabetic retinal impairment and published observations. The general trend of decreasing AV difference was reported by Hammer et al.,^1^ Jørgensen et al.,^4^ Veiby et al.,^6^ and Šínová et al.^5^ However, Hardarson and Stefánsson^2^ and Khoobehi et al.^3^ had not observed a statistical significance of this trend. Moreover, these abovementioned studies have only observed decreasing of AV difference in correlation with progression of NPDR. Further reduction of AV difference with progression from severe NPDR to PDR have not been proven at all. Furthermore, from a paper by Hammer^1^ and Veiby^6^ there seems to be an opposite trend after the transition from severe NPDR to the proliferative form, with the observation of an actual rise in the AV difference. However, none of those authors have proven statistically a significant variance in AV difference between severe NPDR and PDR group.

Changes in saturation in retinal veins and AV difference are considered to be related to the obliteration of some retinal capillaries and consequent bypassing of these areas by highly saturated arterial blood.^2^ It is our opinion that the increase of saturation in retinal veins and the corresponding decrease of AV difference could possibly represent the expression of a degree of general retinal hypoxia in diabetic patients.

Increased saturation in retinal arterioles has also been observed in several previous cross-sectional studies.^1^^–^^7^ Various explanations have been proposed for this phenomenon, such as thickening of vessel walls and reduced counter-current exchange of oxygen between arterioles and nearby venules. Previous articles have also mentioned the possibility of faster retinal blood flow in diabetic patients, which would allow less oxygen to escape from per unit volume of blood through the walls of retinal arterioles.^14^

In the present study we observed a higher level of both proangiogenic factors VEGF and PlGF in the vitreous fluid of diabetic patients with DR, compared to nondiabetic patients, where these factors were below the detectable limit in most cases. Even though the PlGF level did not reach a statistical significance (*P* = 0.0552), it is in good agreement with previous published findings.^15^ Interestingly, trend of higher vitreous levels of antiangiogenic PEDF (*P* = 0.0517) were also observed in our diabetic cohort. Elevated concentrations of PEDF in the present study contradict the findings of a number of other studies that have shown an increase in VEGF and associated decrease in PEDF in the vitreous of patients with PDR.^16^^–^^18^ On the other hand, similar findings to ours were reported by Chernykh et al.^19^ As they hypothesized, the increase in PEDF can be considered a compensatory mechanism aimed at reducing angiogenesis related to the duration of DR. Moreover, patients with proliferative DR where PEDF decrease has been found, formed only a part of our DR cohort, and this could be one of the possible explanations of our findings. This highlights some ambiguities in the physiological effects of PEDF and requires further investigation.

The most interesting finding in our current study is that AV differences correlate with vitreous level of proangiogenic factors (VEGF and PlGF) in diabetic patients. This finding supports the concept that retinal oximetry is a reasonable tool for monitoring severity of DR. To the best of our knowledge, this is the first study focusing on the relationship between the degree of hypoxia measured by automatic retinal oximetry and the intravitreal level of growth factor involved in DR pathogenesis.

A recent study by Jeong et al.^20^ found significant correlation between hypoxia expressed by the ischemic index and proangiogenic factors (VEGF and PlGF). Although the proangiogenic factors were measured in the aqueous fluid, there is strong support in the literature that aqueous and vitreous levels are highly correlated.^21^

The first study focusing on the implications of ischemic status in DR patients was performer by Guduru et al.^7^ They found that oxygen saturation in retinal arterioles and venules increased with DR severity, similar to our current findings. Moreover, saturation in retinal arteries correlates with increasing ischemia measured by wide-field fluorescein angiography. In retinal veins, such correlation was not found, but appeared to indicate a trend. Our study complements and supports this finding using different methods. In our study severity of hypoxia expressed by VEGF production correlates the most with the AV difference. Retinal vein saturation shows only a trend in such correlation (*P* = 0.069) and arterial saturation did not correlate with VEGF level at all. These findings could support explanation that an increase of arterial saturation, as described in previous studies,^1^^–^^7^ is probably related more to blood flow velocity then to degree of retinal hypoxia itself.^15^ PlGF production in vitreous shows a similar trend regarding correlation with retinal hypoxia, as we observe in the vitreous VEGF level in our study. This indicates a synergic effect of VEGF and PlGF.^16^

Our findings and overall consensus in the literature suggest that retinal oximetry could be used for monitoring of DR severity with regard to the degree of hypoxia and the related level of VEGF expression. Our current results moreover indicate that the relationship is not merely a linear function, but seems to be exponential, as can be surmised from the correlation between log VEGF level and AV difference. However, these results need to be interpreted very carefully because of the pilot character of our current study. One of the hypothetical explanations could be the disruption of the retinal-blood barrier in more advanced cases of proliferative DR. In such cases, there is a high suspicion of vitreous sample contamination by growth factors from the blood, resulting in artifactual increase of growth factor levels in the vitreous. However, because of an image quality cutoff, a fresh, dense vitreous hemorrhage would be automatically excluded from the final analysis and likely prevent this error. We do recognize that further research is needed in this field.

On the other hand, the PEDF levels in the vitreous did not show any correlation with oxygen saturation values in retinal vessels in our study. This finding indicates that PEDF expression might not be primarily regulated by hypoxia, but other mechanisms could also be involved.^22^ With respect to the study, the findings should be interpreted in the context of the following limitations. The study sample size was small, and only patients suitable for invasive procedures were recruited (PPV or intravitreal injection) for ethical reasons. Based on this limitation there was an age difference between diabetic patients and the control group (composed of ERM and MH patients). Moreover, an equal representation of all stages of DR was not possible in the diabetic group (there were few cases of DM without DR, DME and PDR were common, and mild and moderate DR were absent).

The effect of laser photocoagulation on retinal oximetry results are not fully elucidated, and studies report conflicting results.^4^^,^^23^ For this reason, we excluded patients after panretinal photocoagulation, and only patients who underwent focal laser treatment were allowed in the study. The effect of laser needs to be the focus of future studies with larger cohorts.

In conclusion, our findings in diabetic patients appear to indicate that there are increased levels of intravitreal VEGF a PlGF as compared to healthy controls and also an increase in oxygen saturation in retinal arteries and veins. Furthermore, our study suggests a correlation between the intravitreal level of proangiogenic factors and the AV oxygen difference as measured by automatic retinal oximetry and this correlation seems to be exponential. However, because of the limitations of our pilot study, future research will be required to better understand and interpret our findings.

## References

[1] Hammer M, Vilser W, Riemer T, et al. Diabetic patients with retinopathy show increased retinal venous oxygen saturation. *Graefes Arch Clin Exp Ophthalmol*. 2009; 247: 1025–1030. 19404666 10.1007/s00417-009-1078-6

[2] Hardarson SH, Stefánsson E. Retinal oxygen saturation is altered in diabetic retinopathy. *Br J Ophthalmol*. 2012; 96: 560–563. 22080478 10.1136/bjophthalmol-2011-300640

[3] Khoobehi B, Firn K, Thompson H, Reinoso M, Beach J. Retinal arterial and venous oxygen saturation is altered in diabetic patients. *Invest Ophthalmol Vis Sci*. 2013; 54: 7103. 24114546 10.1167/iovs.13-12723

[4] Jørgensen CM, Hardarson SH, Bek T. The oxygen saturation in retinal vessels from diabetic patients depends on the severity and type of vision-threatening retinopathy. *Acta Ophthalmol (Copenh)*. 2014; 92: 34–39. 10.1111/aos.12283 24330421

[5] Šínová I, Chrapek O, Mlčák P, Řehák J, Karhanová M, Šín M. Automatic retinal oxymetry in patients with diabetic retinopathy. *Czech Slovak Ophthalmol*. 2016; 72: 182–186. 28224804

[6] Veiby NCBB, Simeunovic A, Heier M, et al. Venular oxygen saturation is increased in young patients with type 1 diabetes and mild nonproliferative diabetic retinopathy. *Acta Ophthalmol (Copenh)*. 2020; 98: 800–807. 10.1111/aos.14462 32410388

[7] Guduru A, Martz TG, Waters A, Kshirsagar AV, Garg S. Oxygen saturation of retinal vessels in all stages of diabetic retinopathy and correlation to ultra-wide field fluorescein angiography. *Invest Ophthalmol Vis Sci*. 2016; 57: 5278. 27723894 10.1167/iovs.16-20190

[8] Šínová I, Řehák J, Nekolová J, et al. Correlation between ischemic index of retinal vein occlusion and oxygen saturation in retinal vessels. *Am J Ophthalmol*. 2018; 188: 74–80. 29366614 10.1016/j.ajo.2018.01.015

[9] Kennedy A, Frank RN. The influence of glucose concentration and hypoxia on VEGF secretion by cultured retinal cells. *Curr Eye Res*. 2011; 36: 168–177. 21158590 10.3109/02713683.2010.521968

[10] Geirsdottir A, Palsson O, Hardarson SH, Olafsdottir OB, Kristjansdottir JV, Stefánsson E. Retinal vessel oxygen saturation in healthy individuals. *Invest Ophthalmol Vis Sci*. 2012; 53: 5433. 22786895 10.1167/iovs.12-9912

[11] Blondal R, Sturludottir MK, Hardarson SH, Halldorsson GH, Stefánsson E. Reliability of vessel diameter measurements with a retinal oximeter. *Graefes Arch Clin Exp Ophthalmol*. 2011; 249: 1311–1317. 21499769 10.1007/s00417-011-1680-2

[12] Hardarson SH, Harris A, Karlsson RA, et al. Automatic retinal oximetry. *Invest Ophthalmol Vis Sci*. 2006; 47: 5011. 17065521 10.1167/iovs.06-0039

[13] Hardarson SH, Basit S, Jonsdottir TE, et al. Oxygen saturation in human retinal vessels is higher in dark than in light. *Invest Ophthalmol Vis Sci*. 2009; 50: 2308. 19117923 10.1167/iovs.08-2576

[14] Jeppesen SK, Bek T. The retinal oxygen saturation measured by dual wavelength oximetry in larger retinal vessels is influenced by the linear velocity of the blood. *Curr Eye Res*. 2019; 44: 46–52. 30230380 10.1080/02713683.2018.1524015

[15] Yu Y, Zhang J, Zhu R, et al. The profile of angiogenic factors in vitreous humor of the patients with proliferative diabetic retinopathy. *Curr Mol Med*. 2017; 17: 280–286. 29110608 10.2174/1566524017666171106111440

[16] Mohan N, Monickaraj F, Balasubramanyam M, Rema M, Mohan V. Imbalanced levels of angiogenic and angiostatic factors in vitreous, plasma and postmortem retinal tissue of patients with proliferative diabetic retinopathy. *J Diabetes Complications*. 201; 26: 435–441. 22699109 10.1016/j.jdiacomp.2012.05.005

[17] Boehm BO, Lang G, Feldmann B, et al. Proliferative diabetic retinopathy is associated with a low level of the natural ocular anti-angiogenic agent pigment epithelium-derived factor (PEDF) in aqueous humor. A pilot study. *Horm Metab Res*. 2003; 35: 382–386. 12920663 10.1055/s-2003-41362

[18] Yokoi M, Yamagishi SI, Saito A, et al. Positive association of pigment epithelium-derived factor with total antioxidant capacity in the vitreous fluid of patients with proliferative diabetic retinopathy. *Br J Ophthalmol*. 2007; 91: 885–887. 17301120 10.1136/bjo.2006.110890 PMC1955675

[19] Chernykh V, Varvarinsky E, Smirnov E, Chernykh D, Trunov A. Proliferative and inflammatory factors in the vitreous of patients with proliferative diabetic retinopathy. *Indian J Ophthalmol*. 2015; 63: 33. 25686060 10.4103/0301-4738.151464 PMC4363955

[20] Jeong A, Yao X, Van Hemert J, Sagong M. Clinical significance of metabolic quantification for retinal nonperfusion in diabetic retinopathy. *Sci Rep*. 2022; 12: 9342. 35665762 10.1038/s41598-022-13439-z PMC9167306

[21] Wu F, Phone A, Lamy R, et al. Correlation of aqueous, vitreous, and plasma cytokine levels in patients with proliferative diabetic retinopathy. *Invest Ophthalmol Vis Sci*. 2020; 61(2): 26. 10.1167/iovs.61.2.26 PMC7326572 32084272

[22] Wang Y, Liu X, Quan X, et al. Pigment epithelium-derived factor and its role in microvascular-related diseases. *Biochimie*. 2022; 200: 153–171. 35661748 10.1016/j.biochi.2022.05.019

[23] Vergmann AS, Torp TL, Kawasaki R, et al. Retinal vascular oxygen saturation in response to a less extensive laser treatment in proliferative diabetic retinopathy. *Acta Ophthalmol (Copenh)*. 2021; 99: 783–789. 10.1111/aos.14727 33354935

